# A mathematical model reveals sex-specific changes in glucose and insulin tolerance during rat puberty and maturation

**DOI:** 10.3325/cmj.2020.61.107

**Published:** 2020-04

**Authors:** Marta Balog, Vedrana Ivić, Rudolf Scitovski, Irena Labak, Kálmán Ferenc Szűcs, Robert Gaspar, Sándor G. Vári, Marija Heffer

**Affiliations:** 1 Department of Medical Biology and Genetics, Faculty of Medicine Osijek, J. J. Strossmayer University of Osijek, Osijek, Croatia; 2 Department of Mathematics, J. J. Strossmayer University of Osijek, Osijek, Croatia; 3 Department of Biology, J. J. Strossmayer University of Osijek, Osijek, Croatia; 4 Department of Pharmacology and Pharmacotherapy, Faculty of Medicine, University of Szeged, Szeged, Hungary; 5 Cedars-Sinai Medical Center, International Research and Innovation in Medicine Program, Los Angeles, CA, United States; The first two authors contributed equally.

## Abstract

**Aim:**

To evaluate the effects of maturation and sex on glucose metabolism during glucose tolerance (GTT) and insulin tolerance tests (ITT) in young and adult male and female rats by using two different approaches – the conventional, which uses area under the curve and glucose curve, and mathematical modeling that identifies parameters necessary for determining the function that models glucose metabolism.

**Methods:**

Male and female rats at 3.5 and 12 months of age underwent standard GTT and ITT after overnight fasting. The parameters were identified by using Mathematica-module NonlinearModelFit [] for experimentally obtained data.

**Results:**

When data were statistically analyzed, both sexes and age groups had similar glucose and insulin tolerance. In the mathematical model of GTT, parameters describing the rate of glucose concentration increase *G’*(0) and decrease *G’_I_* multiplied with maturation, with a concomitant decrease in the time point (*t_max_, t_I_*) of reaching maximum and minimum glucose concentration (*G_max_, G_0_*). The mathematical model of ITT for males was independent of age, unlike that for females, which had increased *G’*(0) and *G’_I_,* and more quickly recovered from hypoglycemia after maturation.

**Conclusion:**

The mathematical model revealed female susceptibility to large glucose excursions, which are better reflected by ITT in young animals and by GTT in adults.

Glucose tolerance test (GTT) and insulin tolerance test (ITT) are common experimental and clinical biochemical tests based on (plasma/serum/whole blood) glucose monitoring during two up to four hours from the base point (1,2). GTT assesses the body's ability to maintain normoglycemia after glucose load. The obtained glucose profile is the result of the action of multiple hormones that have similar (insulin/leptin), opposite (insulin/glucagon), or mutually modulating (insulin/incretins) effects (3,4). ITT assesses the stress response to insulin bolus. A steep drop in insulin-induced hypoglycemia excites the hypothalamic-pituitary-adrenal axis, finally leading to the secretion of two hormones – adrenaline and cortisol (corticosterone in rodents) – which oppose insulin action and re-establish normoglycemia (5,6). Glucose homeostasis depends on insulin release from β-cells of Langerhans islets of the pancreas and the sensitivity of the target tissues to insulin (ie, glucose storage and expenditure by the target tissues). The relationship between these two factors determines the total physiological tolerance of an individual to glucose and the individual's ability to maintain glucose homeostasis. GTT reflects glucose sensitivity and can be performed by various methods – quantifying the insulin release from β-cells as a response to plasma glucose concentration, determining the target tissues' sensitivity to insulin, and determining glucose tolerance, which is informative of both – insulin release from β-cells and target tissues sensitivity to insulin (1,7,8). On the other hand, ITT reflects insulin sensitivity, primarily in the liver and skeletal muscles (3,9). In clinical setting, fasting blood glucose (FPG) and 2-h plasma glucose (2-h PG) during 75 g oral glucose tolerance test (OGTT) are used to diagnose prediabetes (FPG 5.6-6.9 mmol/L, 2-h PG 7.8-11.0 mmol/L) and diabetes (FPG≥7 mmol/L, 2-h PG≥11.1 mmol/L) (10). However, FPG and final point of OGTT often miss patients with impaired glucose tolerance (IGT) and impaired fasting glucose (IFG), two intermediate states leading toward adult-onset diabetes or type 2 diabetes mellitus (T2DM). Since there are more than eight identified molecular mechanisms contributing to postprandial hyperglycemia and more than 143 common genetic variants associated with T2DM, IGT and IFG only crudely represent the heterogeneous glycemic response and its impairment (11,12). More prediabetic subphenotypes are expected to be classified by a closer analysis of glucose peaks (connected with cardiovascular risk) and overall glycemic variability. ITT is less frequently performed for clinical purposes because there is a risk of adverse effects (sweating, palpitations, epilepsy, cardiac ischemic event, etc) and the outcome cannot be interpreted unless hypoglycemia below 2.2 mmol/L is achieved (13). Nevertheless, the test is considered the gold standard for diagnosing hypopituitarism and Cushing’s syndrome (14). ITT is more often performed on experimental animals to test their ability to maintain metabolic balance under stressful conditions (15,16). Together, GTT and ITT reflect a number of molecular mechanisms responsible for normoglycemia – the prerequisite for the function of all organs, especially the brain.

While maturation is the sharpening of biological response, aging is a decay characteristic for biological entities, described as gradual degeneration rather than sudden collapse (17). Progressive degeneration of molecular mechanisms maintaining normoglycemia is reflected in glucose profile and easily accessible by GTT and ITT (18). The identification of the currently missing mathematical parameters describing the dynamics of glycemic profile in both tests can indicate subtle changes, point out impaired molecular mechanisms, and lead toward personalized therapy.

The hypothesis of this study is that the interpretation of GTT and ITT test by classical calculation and statistics at the level of area under the curve (AUC) cannot detect metabolic differences between young and adult or male and female rats, whereas newly identified parameters describing the mathematical equation that had been fitted to experimental data pinpoint and quantify the differences between studied groups. The first aim of this study was to analyze the acquired data from GTT and ITT using mathematical modeling in order to reveal the details of glucose dynamics. The second aim of the study was to evaluate the influence of maturation and sex on glucose metabolism.

## MATERIAL AND METHODS

**Figure 1:**
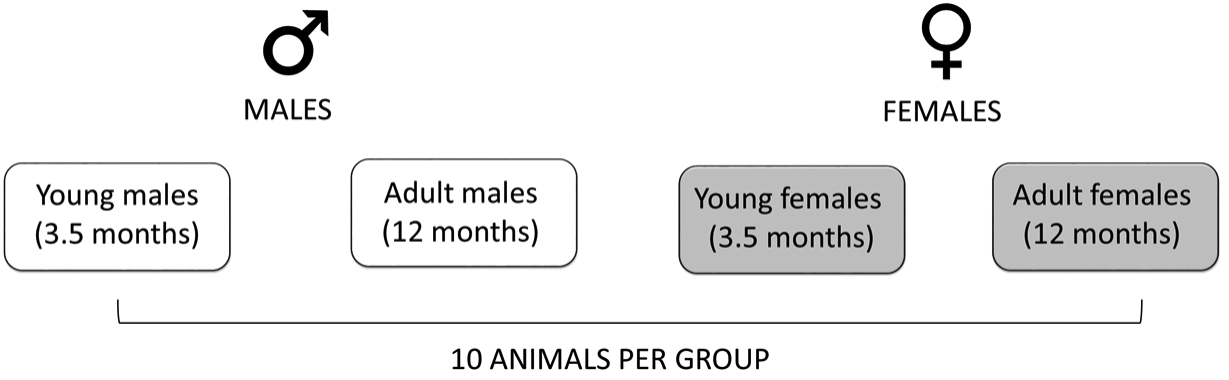
Study design – classification of the animal groups.

The study, conducted from May to December 2016, used Sprague Dawley-CR rats (Charles River, Sulzfeld, Germany). In total, 40 animals were used: 10 young males and females, 3.5 months old, and 10 adult males and females, 12 months old (Figure 1). The experiment on young animals was performed at the Faculty of Medicine, J. J. Strossmayer University of Osijek, Croatia and the experiment on adult animals was performed at the University of Szeged, Hungary. Both parts of the study were executed with the same equipment and chemicals. The study was approved by the Croatian Ministry of Agriculture (2158-61-07-14-118) and National Scientific Ethics Committee on Animal Experimentation of Hungary (IV/3796-7/2015). The rats were kept in self-ventilating cages with housing temperature between 21°C and 24°C, five air changes per minute, and constant humidity of 40%-60% (THF3364, Ehret, Freiburg, Germany). There were maximum three young animals per cage and at least two adult rats per cage. Standardized food for experimental rats (4RF21, Mucedola, Milan, Italy) and tap water were available *ad libitum* except 12-14 hours before GTT and 3 hours before insulin ITT. Day cycle was set to 7.00 am-7.00 pm

### Glucose and insulin tolerance tests

Peritoneal GTT was performed in all animals after 12-14 hours of fasting by injecting 2 g of glucose/kg of body mass in the peritoneum. Glucose (Merck, Branchburg, NJ, USA) was dissolved in distilled water as a 25% working solution. Animals were heated with infrared lamp (R95E, Philips, Amsterdam, the Netherlands) for easier blood sampling from the lateral tail vein. Glucose was measured at 8 time points for GTT and ITT.

GTT was carried out as follows:

1. Rats fasted for 12-14 hours.

2. Basal glucose concentration was measured by tail puncture.

3. Glucose was injected intraperitoneally.

4. Fifteen minutes after glucose injection the second measurement of glucose concentration was performed.

5. The measurement of glucose concentration was continued after 30, 45, 60, 90, 120, and 240 minutes from glucose application.

6. At the end of the test, the animals were given food.

Peritoneal ITT was performed after 3 hours of fasting by injecting 0.75 U/kg of insulin Humalog (Lilly, Indianapolis, IN, USA) in the peritoneum. Animals were heated with infrared light for easier blood sampling from the tail vein. Glucose was measured at 8 time points: 0, 15, 30, 45, 60, 90, 120, and 180 minutes.

ITT was carried out as follows:

1. Rats fasted for 3 hours.

2. Basal glucose concentration was measured by tail puncture.

3. Insulin was injected intraperitoneally.

4. Fifteen minutes after glucose injection the second measurement of glucose concentration was performed.

5. The measurement of glucose concentration was continued after 30, 45, 60, 90, 120, and 180 minutes from insulin application.

6. At the end of the test, the animals were given food.

In both tests, the glucose concentration from a drop of blood (approximately 50 μL of blood per animal) was measured by the OneTouch Ultra Mini glucometer (Life Scan, Milpitas, CA, USA) as follows:

1. The animal was put in an appropriate plastic cylinder holder, and the tail was heated with an infrared lamp.

2. The tail was wiped with ethanol.

3. An appropriate needle was injected under the skin and into the lateral tail vein to obtain a drop of blood for measurement; aspiration was not performed due to possible vein collapse.

4. A drop of blood was applied to the glucose strip, inserted into the glucometer, and glucose concentration was read as mmol/L.

### Statistical analysis

AUCs were calculated and compared by using Statistica 12 software (Tibco, Palo Alto, CA, SAD). Normality of distribution was tested by Shapiro-Wilk test. Mann-Whitney test with Bonferroni correction was applied to determine the significance of differences between AUCs of different animal groups. The level of significance was set to <0.05.

### Development of mathematical model for glucose and insulin tolerance tests

The mathematical model was developed based on the following logic: let us consider the concentration of glucose *G* in blood and the net normal hormonal concentration *H* as a cumulative effect of all relevant hormones (for example, insulin and leptin decrease *G*, while cortisol and growth hormone increase *G*). A basic model can be written according to (19,20): 
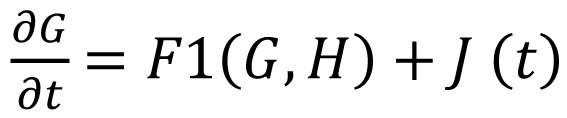
, 
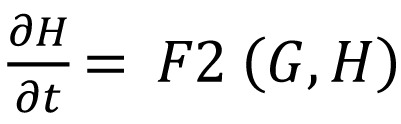


The function J is the external rate at which the blood glucose concentration is increased due to absorption rate. We assume that the quantities *G* and *H* attain optimal values *G_0_* and *H_0_* at the point when the patient arrives to hospital on an empty stomach. By using the substitution g = *G* − G_0_ and *h* = *H* − *H_0_* and Taylor’s theorem, we obtain a system of linear differential equations for the functions g and *h*: 
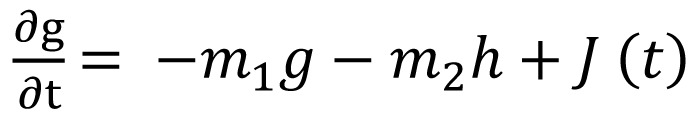
, 
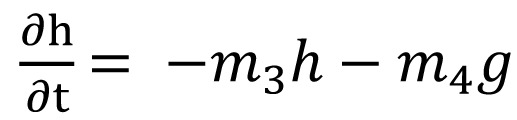


where *m_1_*, *m_2_*, *m_3_*, *m_4_* are positive constants. It can be shown that the functions *g* and *h* satisfy linear differential equation of the second order with constant coefficients:


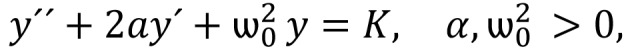


where 
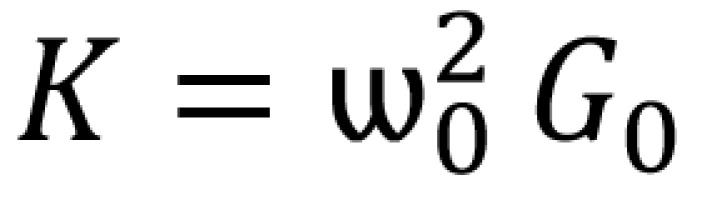
 for the function *g* and 
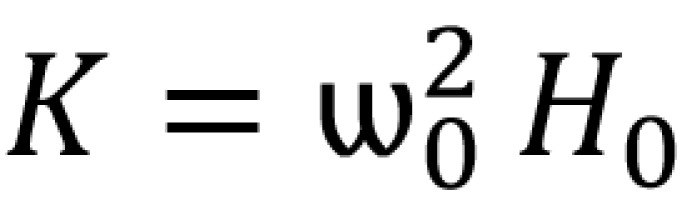
 for the function *h*. If experimental data (*t_i_, y_i_*), *i* = 1, *…, m* are known, where *t_i_* is the time point of measuring glucose *G_i_* concentration, we can estimate the parameters in the above stated model by solving the corresponding parameter identification problem (21-24). For solving this problem we used Mathematica-module NonlinearModelFit [] (25). In this way we obtained a good approximation of parameters α, 
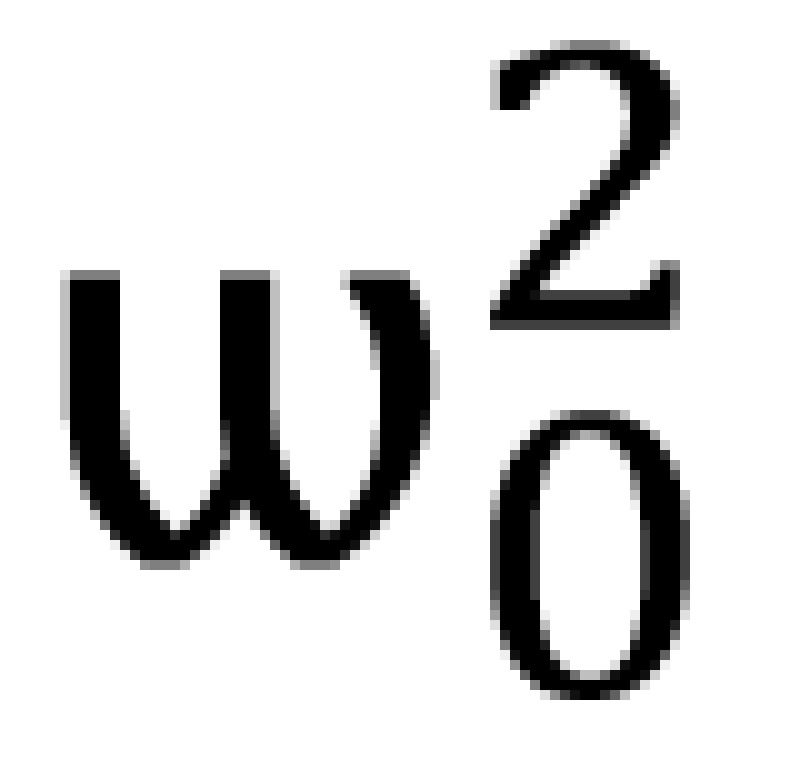
, and *G*_0_ in the differential equation and optimal initial condition μ = *y* (0) and ν = *y*´ (0) in the corresponding Cauchy´s problem. In the case of glucose concentration, by knowing the parameters α, 
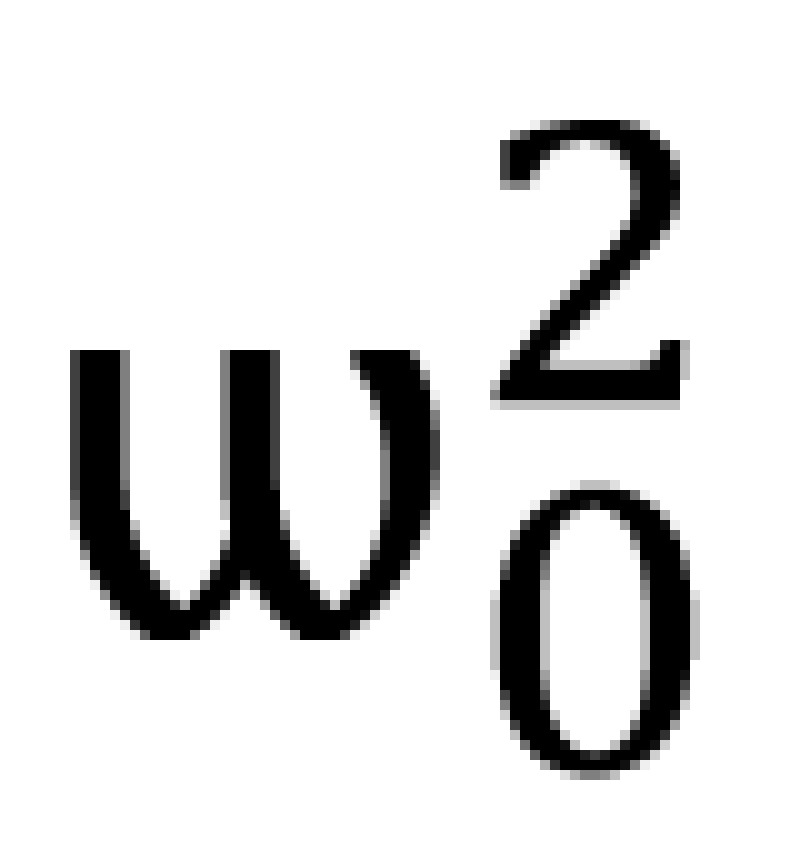
,and *G*_0_ in the differential equation and optimal initial condition *G*(0) and *G*´(0), we can write the required function *G* as the solution to the corresponding Cauchy´s problem, whereby the crucial role belongs to the corresponding characteristic equation:


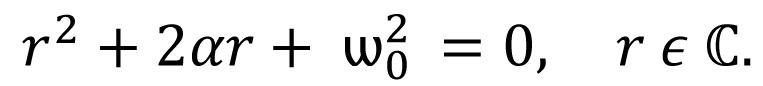


If 
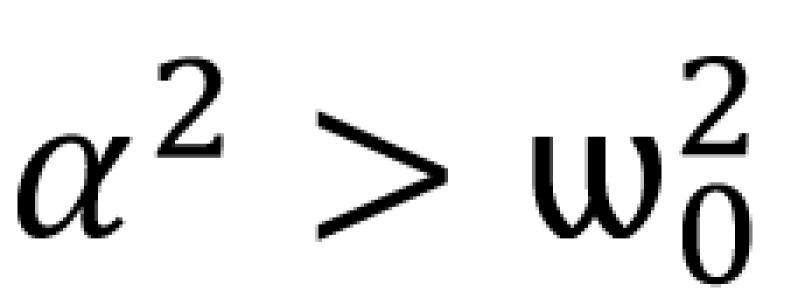
, then the roots *r*1, *r*2 of the characteristic equation are negative and mutually different real numbers, and the solution of the Cauchy´s problem for the differential equation is obtained in the form 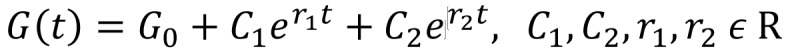. If >
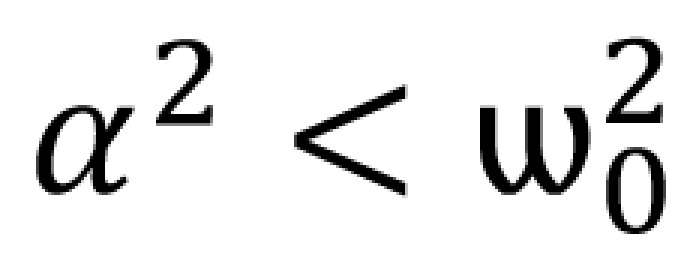
, the roots *r*1, *r*2 of the characteristic equation are conjugate complex numbers:


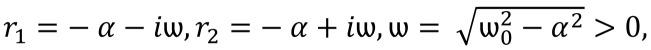


and the solution of the Cauchy´s problem for the differential equation is obtained in the form:


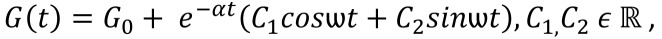


or in the form:


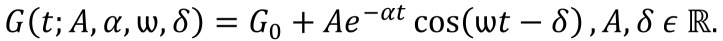


**Figure 2:**
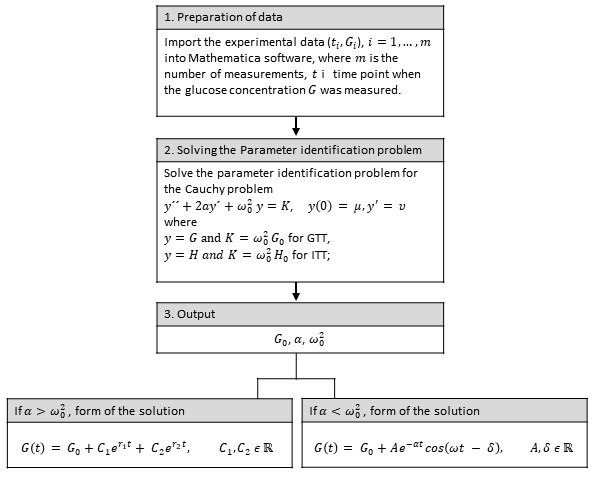
Workflow diagram for determining the function modeling blood glucose.

The workflow of determining the function G is depicted in Figure 2. The characteristics of this function are initial glucose concentration *G*(0), initial rate of glucose absorption *G*´(0), maximum glucose concentration *G_max_,* which is achieved at the moment *t_max_*, maximum speed of glucose concentration decrease *G´_I_* (a negative number because it represents the decrease of concentration), the moment *t_I_* (inflexion) in which the maximum speed of glucose concentration decrease is obtained, stabilized glucose concentration 
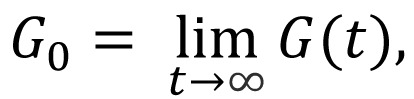
 AUC, and estimated variance (EstVar). The same applies both to changes in blood glucose concentration under the influence of glucose load (GTT) or insulin bolus (ITT).

To evaluate the overall difference between real data and the values predicted by the mathematical model we calculated residual sum of squares and R^2^ (determination coefficient).

## RESULTS

### Classical analysis: young and adult rats of both sexes are similarly exposed to hyperglycemia during GTT or hypoglycemia during ITT test

**Figure 3:**
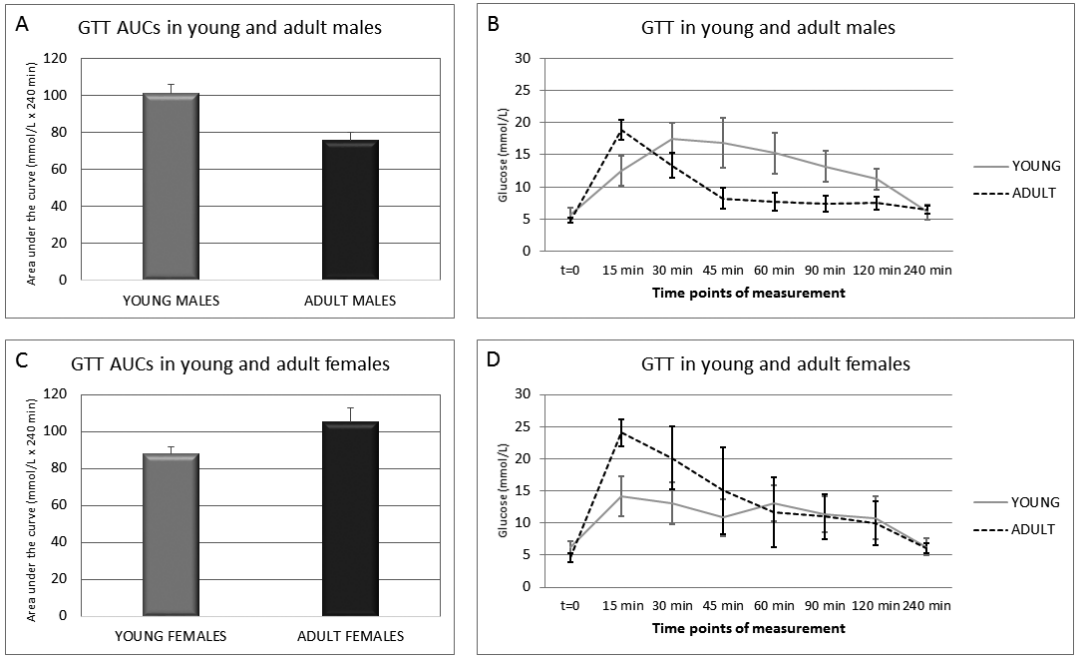
Glucose tolerance test (GTT) in young and adult male and female Sprague Dawley rats. (**A**) Areas under the glucose curve (AUCs) of young and adult males. (**B**) Glucose curve of young and adult males. (**C**) AUCs of young and adult females. (**D**) Glucose curve of young and adult females. Glucose curves and AUCs were constructed using trapezoidal rule based on experimental data. Mann-Whitney test with Bonferroni correction was applied for determination of significant differences between AUCs of different animal groups, p value was set to <0.05. No significant differences were determined.

GTT data were analyzed by classical comparison of AUCs (Figure 3A and B). In males, AUC decreased after maturation (Figure 3A). Unlike young males, adult males reached maximum glucose concentration very fast and returned to normal glucose concentration range after 45 minutes (Figure 3B). In females, AUC increased after maturation (Figure 3C). However, in young females the glucose curve was biphasic (Figure 3D) – it reached the first glucose concentration peak after 15 minutes and the second peak after 60 minutes of the test. In adult females the glucose curve was not biphasic, and it reached maximum glucose concentration after 15 minutes. Because the differences between young and adult animals of both sexes were not significant, we concluded that glucose tolerance during maturation remained similar.

**Figure 4:**
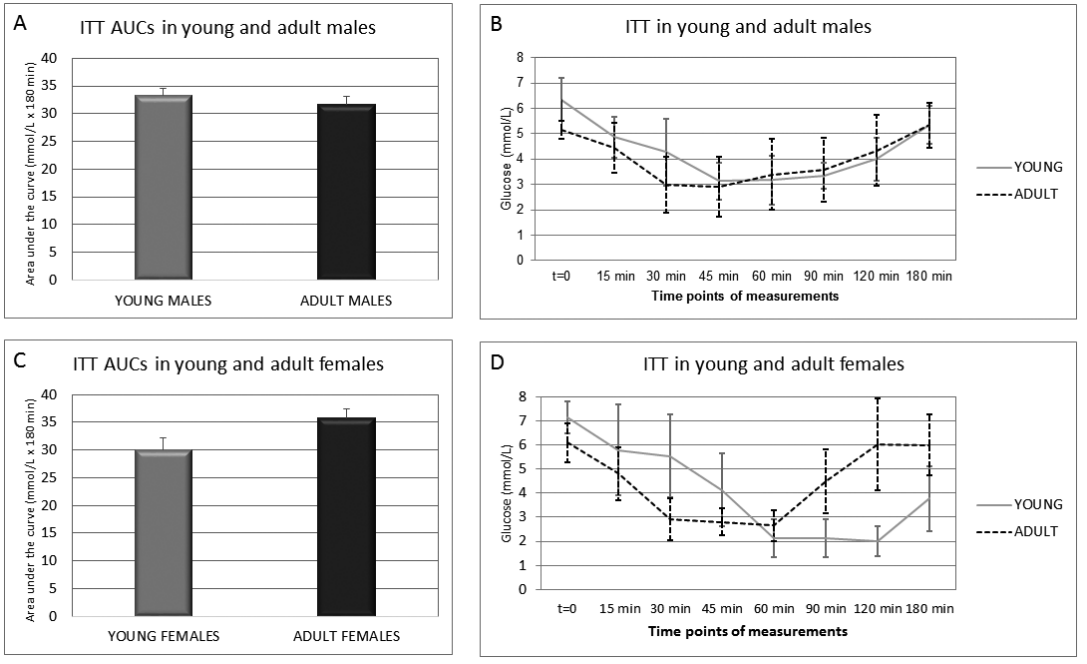
Insulin tolerance test (ITT) in young and adult male and female Sprague Dawley rats. (**A**) Areas under the glucose curve (AUCs) of young and adult males. (**B**) Glucose curve of young and adult males. (**C**) AUCs of young and adult females. (**D**) Glucose curve of young and adult females. Glucose curves and AUCs were constructed using trapezoidal rule based on experimental data. Mann-Whitney test with Bonferroni correction was applied for determination of significant differences between AUCs of different animal groups, p value was set to <0.05. No significant differences were determined.

AUCs for ITT were the same in young and adult males (Figure 4A). Young males reached the minimum glucose concentration later (after 45 minutes) than adult males, who reached the minimum glucose concentration after 30 minutes of the test (Figure 4B). Both male animal groups returned to glucose concentration slightly above 5 mmol/L after 180 minutes of the test, which is a sign of normoglycemia (defined as glucose concentration between 4.2 and 5.5 mmol/L). Adult females had higher AUC than young females (Figure 4C). Young females became hypoglycemic after 60 minutes and maintained such low glucose concentration until 120 minutes of the test (Figure 4D) and did not become normoglycemic even after 180 minutes of the test. Conversely, adult females reached hypoglycemic glucose concentration after 60 minutes and returned to normoglycemia between 90 and 120 minutes of the test. The statistical analysis of AUCs for both GTT and ITT revealed no significant differences inside same sex groups or between the two age groups. Because observed differences between young and adult animals of both sexes were not significant, we concluded that insulin sensitivity during maturation also remained similar.

### Mathematical model: glucose dynamics is age and sex specific

**Figure 5:**
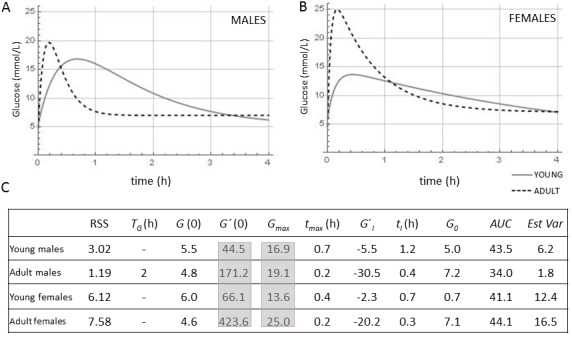
Mathematical model of glucose tolerance test (GTT) in young and adult male (**A**) and female (**B**) Sprague Dawley rats. (**C**) The properties identified by the mathematical model: AUC = area under the curve, *EstVar* = estimated variance, *G*(0) = fasting glucose, *G´*(0) = initial rate of glucose concentration increase, *G_0_* = stabilized glucose concentration, *G´_I_* = maximum rate of glucose concentration decrease, *G_max_* = maximum concentration of glucose, *T_G_*(h) = basic period of function *G*, *t_I_* = time point in which *G´_I_* was reached, *t_max_* = time point in which *G_max_* was reached. Residual sum of squares (RSS) was calculated to evaluate the quality of mathematical modeling, and data are shown in the table. The most discriminatory mathematical parameters between animal groups are shaded in gray.

When mathematical modeling was applied to the same GTT data, the rate of glucose increase, *G´*(0), was almost four times lower in young males than in adult males (Figure 5A and C). *G*_max_ was 13% higher in adult males than in young males, while the rate of glucose decrease, *G´_I_* was six times lower in young than in adult males. The maximum rate of glucose decrease to normoglycemia, *t_I_,* was reached sooner in adult (after 23.1 minutes) than in young males (after 82.0 minutes). Stabilized glucose concentration, *G_0_,* in males increased upon maturation.

The same findings were observed in just 3 out of 10 females that fit to the mathematical model: *G´*(0) was six times, while *G´_I_* was ten times lower in young compared with adult females (Figure 5B and C). Surprisingly, adult females had almost twice as high *G_max_* than young females. In adults, *t_I_* was reached sooner (after 19.6 minutes) than in young females (after 44.3 minutes). *G_0_* in females was also increased upon maturation.

Between sexes, *G´*(0) was 1.5 times higher in young females than in young males and 2.5 times higher in adult females than in adult males. *G´_I_* was 2.4 times higher in young males than in young females and 1.5 times higher in adult males than in adult females. The maximum rate of glucose decrease to normoglycemia, *t_I_,* was almost double in young males (after 82 minutes) than in young females (after 44.3 minutes) and 1.2 times higher in adult males (after 23.1 minutes) than in adult females (after 19.6 minutes). Generally, the rate of glucose increase was lower in males than in females, contrary to the rate of glucose decrease. Stabilized glucose concentration, *G_0_,* was seven times higher in young males than in young females, while the same trend was not observed in adult animals. AUCs predicted by modeling were very similar in both young and adult males and females. The described peculiarities of GTT mathematical modeling were missed by classical AUC and glucose curve. Residual sum of squares in the case of GTT was lowest in males (Figure 5), while R^2^ was over 90% for all the groups (data not shown).

**Figure 6:**
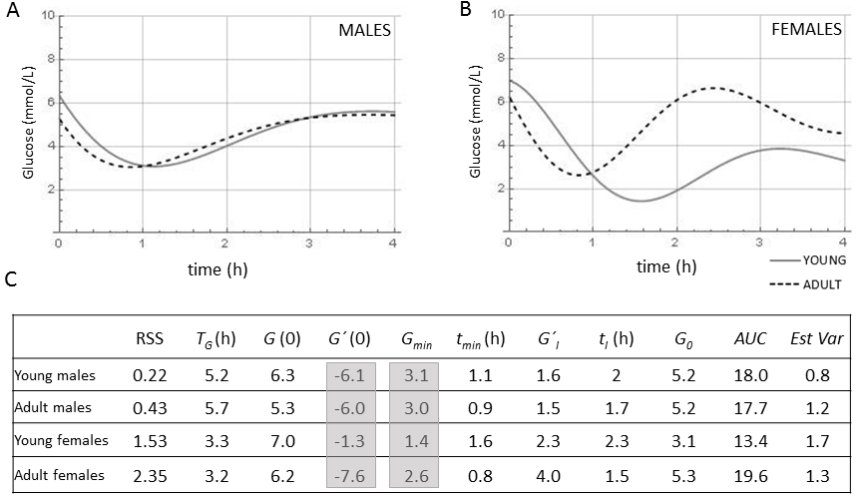
Mathematical model of insulin tolerance test (ITT) in young and adult male (**A**) and female (**B**) Sprague Dawley rats. (**C**) Properties identified by the mathematical model: AUC = area under the curve, *EstVar* = estimated variance, *G*(0) = fasting glucose, *G´*(0) = initial rate of glucose concentration decrease, *G_0_* = stabilized glucose concentration, *G´_I_* = maximum rate of glucose concentration increase, *G_min_* = minimum concentration of glucose, *T_G_*(h) = basic period of function *G*, *t_I_* = time point in which *G´_I_* was reached, *t_min_* = time point in which *G_min_* was reached. Residual sum of squares (RSS) was calculated to evaluate the quality of mathematical modeling and data are shown in the table. The most discriminatory mathematical parameters between animal groups are shaded in gray.

In the case of ITT modeling, the function of young males was very similar to that of adult males (Figure 6A and C). In males, differences were observed only in *t_I_*, which was 16.5% lower in adult than in young males, while the rest of the parameters were almost the same. *G´*(0) was seven times higher in adult females than in young females (Figure 6B and C). The rate of increase to normoglycemia, *G´_I_,* was 1.7 times higher in adult females, therefore *t_I_* was reached sooner in adult (after 91.5 minutes) than in young females (after 135.7 minutes). Stabilized glucose concentration, *G_0_,* decreased 1.7 times in young females.

Between sexes, *G´*(0) was almost five times higher in young males compared with young females, while in adult animals no dramatical differences were observed. Young females developed life-threatening hypoglycemia (*G_min_*), while both age groups of males stayed above the level of hypoglycemia. *G´_I_* was 1.4 times higher in young females compared with young males and 1.7 higher in adult females compared with adult males. The *t_I_* was reached faster in young males (after 122.6 minutes) than in young females (after 135.7 minutes), while the opposite was observed for adult females (after 91.5 minutes) and males (after 102.4 minutes). *G_0_* was decreased in young females compared with young males, while adult males and females had a similar value of *G_0_*_._ AUCs predicted by modeling were very similar in all animal groups except in young females, which had the lowest AUC value. As in GTT modeling, the described peculiarities of mathematical modeling were not observed when ITT results were subjected to classical analysis. Residual sum of squares in the case of ITT was lowest in males (Figure 6), while R^2^ ranged from 86%-99% for all animal groups (data not shown).

## DISCUSSION

Using mathematical modeling, we generated new parameters that described GTT and ITT results in greater detail than the AUC approach. The following mathematical parameters were more discriminatory: *G´*(0) – the initial rate of glucose concentration increase (for GTT) or decrease (for ITT), *G_max_* – maximum glucose concentration for GTT, *G_min_* – minimum glucose concentration for ITT, *t_I_* – the time point when *G´_I_* was reached, *t_max_* – the time point when *G_max_* was reached in GTT, and *t_min_* – the time point when *G_min_* was reached in ITT. Residual sum of squares and R^2^ revealed whether modeled curve fitted real data – residual sum of squares was not ideal and overall it was lower in males than females. R^2^ revealed perfect fit (86%-99%), but this finding should be interpreted with caution since our data did not satisfy all theoretical properties for its usage due to small sample size and nonlinearity of the model.

When the AUCs were compared between classical data (Figure 3 and 4) and modeled data (Figure 5 and 6), the differences in glucose and insulin tolerance between the groups were not obvious, meaning that in both cases AUC is not the ideal parameter describing the glucose dynamics.

GTT modeling revealed that adult females reached *t_max_* faster and had higher maximum glucose concentration (*G_max_*) as well as slower return to normoglycemia, which implies a possible metabolic disbalance upon such glucose fluctuation. The modeled AUC for GTT was lowest in adult males, revealing their good tolerance to glucose. ITT modeling revealed more prominent differences in young females – *G´*(0) and *G_min_* were lowest and hypoglycemia lasted longer than in other groups. The predicted AUC for ITT was evidently lower in young females, which indicates their susceptibility to hypoglycemia and implies slower gluconeogenesis response. Both young and adult male and female animals had overall good tolerance to glucose and insulin, however their glucose dynamics was different during GTT and ITT, which became more obvious only after modeling.

Animal models investigating type-2 diabetes and insulin resistance use a wide range of species, mostly rodents, and subject them to genetic, chemical, or nutritional interventions (26-29). It was shown that 16 and 20-month-old C57BL/6J male mice had significantly lower AUC for GTT than 4-month-old mice. The same study revealed that adult males' AUC was slightly reduced in response to insulin compared with that of young males (30). These findings agree with our data; however, the previous study did not quantify glucose dynamics.

Also, age should be taken into account when studying glucose metabolism, since glucose metabolism is influenced by the fine tuning of many hormones at prepubertal and pubertal age. Young rats used in this study match 9-10 year-old humans, while adult rats match 25-30 year-old humans (31).

A study in boys and girls without diabetes showed that physiologic insulin resistance started in prepuberty or puberty (32). Physiologically normal individuals of this age more slowly reach *G_max_* and more slowly return to normoglycemia. Two possible mechanisms underlie such glucose dynamics. During an early stage in sexual maturation, termed adrenarche, adrenal gland hormones are intensively synthesized, especially dehydroepiandrosterone sulfate (DHEA-S) (33). DHEA-S levels are inversely related to insulin sensitivity. The same clinical study also showed that preadrenarchal children were more insulin sensitive than children who already reached adrenarche. Adrenarche occurs earlier in girls than in boys, a factor that may be responsible for the sex-specific insulin sensitivity (34), also observed in our study. Another factor influencing insulin resistance during puberty is an increased secretion of growth hormone (GH), which opposes insulin (35). GH release is stimulated by hypoglycemia and suppressed by oral glucose administration, which presents the standard test for inhibitory control of GH release (36). Young female Sprague Dawley rats had higher GH than males (37), which could explain hypoglycemia in young females observed in our study during ITT. During maturation females undergo different metabolic changes than males due to different reproductive function, so it is reasonable to study the sexes separately. Our results clearly imply that glucose and insulin tolerance changes dramatically with extensive growth, particularly in females. Sexual dimorphism in energy expenditure is an evolutional adaptation that preserves the reproductive function in females during fasting, while males mobilize energy stores during physical activity (38). Young females do not switch to gluconeogenesis as quickly as males, probably due to differences in gluconeogenesis regulation and lipolysis activation. Male metabolism favors gluconeogenesis and the use of glycogen stores, while female metabolism relies on fats during increased physical activity (39). In females free fatty acids are stored in subcutaneous adipose tissue, while in males free fatty acids are oxidized rather than stored (40,41), a finding that is supported by the lower basal fat oxidation in women than in men (42). Insulin is known to suppress lipolysis, ie, the release of free fatty acids after a meal (43). After lipolysis of adipose tissue, women have higher delivery of free fatty acids to the liver, which puts them at a greater risk for hepatic insulin resistance (44,45).

Glucose metabolism has a different dynamics during adolescence than in the adulthood. Upon glucose peritoneal challenge, glucose is absorbed to the blood via peritoneal mesothelial cells. In our study, young females had delayed glucose increase. Similar differences in glucose dynamics between the sexes have been observed when it came to the expression and function of molecular water channels, aquaporins. When 8-10 week-old C57BL/6 mice were exposed to glucose dialysate, males and females had a matching peritoneal delivery rate of low weight molecules, but females had lower aquaporin 1 mRNA in peritoneal mesothelial cells (46). Another *in vitro* study found that the expression of aquaporin 1 in human peritoneal mesothelial cells was significantly increased by glucose exposure (47).

The observed and existing data indicate that adolescents have different glucose metabolism than adults. Young animals are more prone to hypoglycemia, which was observed in females in ITT. Different glucose metabolism in young males and females is caused by different lipid metabolism and different expression of peritoneal water channels and increased GH. Even though the classical glucose curves and AUCs imply reduced glucose and insulin tolerance, this state is caused by normal maturation and does not represent a pathology. Therefore, young animals are not a good model for translational diabetes studies. Also, sex seems to be of a great importance and we strongly believe the sexes should be analyzed separately.

Our study was limited by the fact that the same animals were not longitudinally studied at several time points of aging. Furthermore, the use of euglycemic-hyperinsulinemic glucose clamp test could have provided a better assessment of insulin sensitivity (48). The used mathematical model was not able to predict the biphasic GTT in several young female rats.

However, the developed mathematical model has two important advantages: the possibility to identify the parameters that relevantly describe the blood glucose dynamics and the ability to quantify subtle changes not visible in classical statistical analysis of AUC and glucose curve. This is why we recommend its use in future studies. We also believe the new model is applicable in the diagnostics of prediabetes.
